# Quality of Life and Bariatric Surgery: Cross-Sectional Study and Analysis of Factors Influencing Outcome

**DOI:** 10.1007/s11695-016-2220-2

**Published:** 2016-05-14

**Authors:** Michał Robert Janik, Tomasz Rogula, Ilona Bielecka, Andrzej Kwiatkowski, Krzysztof Paśnik

**Affiliations:** 1 Department of General, Oncologic, Metabolic and Thoracic Surgery, Military Institute of Medicine, Szaserów, Warszawa, 04-141 Poland; 2 Bariatric and Metabolic Surgery - Geauga Medical Center, Case Western Reserve University School of Medicine, 10900 Euclid Ave. T402, Cleveland, 44106 OH USA

**Keywords:** Obesity, Bariatric surgery, Quality of life, Laparoscopic sleeve gastrectomy, Laparoscopic Roux-en-Y gastric bypass, Confounders

## Abstract

**Purpose:**

The aims of our study were to compare quality of life (QOL) in obese patients after bariatric surgery with that in controls seeking surgery and to investigate which factors are associated with QOL in the Moorehead–Ardelt Quality of Life Questionnaire II (MA II).

**Materials and Methods:**

This was a cross-sectional study. The operated group consisted of patients after laparoscopic sleeve gastrectomy or laparoscopic Roux-en-Y gastric bypass. The MA II was administered by e-mail to 305 patients 12–18 months after surgery. The control groups consisted of 101 obese patients. We compared the QOL scores and considered good and very good outcomes to be satisfactory. Multiple logistic regression and correlation analysis was performed to identify factors associated with QOL.

**Results:**

In the operated group, the total MA II score was 1.70 ± 0.76, which was higher than 0.59 ± 1.17 in the control group. The score adjusted for the type of surgery was comparable. The prevalence of satisfactory QOL outcomes was similar in both post-operative subgroups and was still higher than in the control group. We identified four factors associated with higher QOL in obese patients. Weight loss was not correlated with total score in MAII.

**Conclusions:**

This study demonstrates that patients after bariatric surgery have a higher score in MA II, which reflects better QOL. The scoring adjusted by type of operation is comparable. QOL among obese patients is dependent on age, gender, history of bariatric surgery, and partnered status. Body mass reduction was not associated with outcome in MAII.

## Introduction

The aim of bariatric surgery is to improve obesity-related comorbidity and quality of life (QOL) by reducing excess weight. Many studies have shown a positive impact of surgical weight loss on QOL [1, 2]. However, limited data are available about which factors influence QOL measured by the Moorehead–Ardelt Quality of Life Questionnaire II (MA II) in obese patients, and which factors may be potential confounders. Bariatric surgery is evolving and trends in the type of procedures performed are still changing. Laparoscopic sleeve gastrectomy (LSG) and laparoscopic Roux-en-Y gastric bypass (LRYGB) are currently the most popular types of bariatric operation [3]. The procedures are equally effective in maintaining weight loss during short-term follow-up but LRYGB seems to be more effective in resolving obesity-related comorbidity [4]. There have been several attempts to compare the procedures in terms of QOL after surgery but the amount of evidence is limited [5–7].

The aims of our study were the following: (1) to compare QOL in obese patients 12–18 months after bariatric surgery to that in controls seeking surgery and (2) to investigate which factors influence QOL outcomes in the MA II in obese patients.

## Material and Methods

The study was cross-sectional and data were collected from two separate groups. The operated group comprised 28 patients at 12–18 months after LSG (post-LSG subgroup) and 30 at 12–18 months after LRYGB (post-LRYGB subgroup). The control group consisted of 101 patients seeking bariatric surgery. The questionnaires were sent via e-mail to the operated group and there was a response rate of 19 %. Preoperative data of responders were extracted from medical records. In the controls, the questionnaires were collected prior to surgery and resulted in a 90 % response rate. All participants met the following criteria for bariatric surgery [8]: BMI >40 kg/m^2^ or >35 kg/m^2^ with comorbidity, for >5 years, and failed conservative treatment for >2 years. Patients were assigned to the type of operation on the basis of baseline BMI, presence of comorbidity, and preference for a sweet-tasting diet. Informed consent was obtained from all participants included in the study. Patients with a history of depression and anxiety disorders were excluded. Patients from control group were not included in the operated group.

### Operative Technique and Post-operative Management

In LSG, 75–80 % of the greater curvature was resected, forming a narrow sleeve-shaped stomach. A 36 French bougie was used to calibrate the sleeve. In LRYGB, the stomach was transected, creating a pouch of 25–30 ml in volume. Then, a gastrojejunal anastomosis was performed using a circular stapler. After that, two loops of the small intestine were stapled side to side, and a jejunostomy was created with a Roux limb length of ~150 cm.

Regardless of the type of surgery, patients underwent a methylene blue solution test intraoperatively, and on post-operative day 1. If there was no leakage detected, an oral diet was resumed. The patients were discharged on post-operative day 2. In general, both procedures were performed laparoscopically with the use of five trocars.

### Measurement of QOL

The groups were asked to complete the Moorehead–Ardelt Quality of Life Questionnaire II (MA II) and the department-specific questionnaire. MA II was introduced by Moorehead et al. in 2003 [9] and is used as a part of the Bariatric Analysis and Reporting Outcome System (BAROS). It is a six-item self-report questionnaire that assesses the patient’s subjective impression of QOL across six areas of general self-esteem, physical activity, social contacts, work satisfaction, sexual pleasure, and focus on eating behavior. Each item is scored from −0.5 to +0.5. The total score ranges from −3 to +3 and defines five outcome groups: poor, very poor, fair, good, and very good. Good and very good outcomes are considered as satisfactory.

The department-specific questionnaire assessed body weight and height, comorbidity, partnered status, place of living, and smoking status. Because of the study design, we did not collect data regarding control weight or weight reduction.

### Statistical Analysis

Analysis was performed using the SAS® software, University Edition (SAS Institute, Cary, NC, USA). To compare continuous variables, the Mann–Whitney *U* and unpaired Student *t* tests were used. Categorical variables were compared using the χ^2^ and Fisher’s exact tests. Statistical significance was set at *p* < 0.05. Multiple logistic regressions with stepwise variable selection were used to construct a model for prediction of satisfactory QOL outcome. Backward stepwise elimination and forward stepwise selection were both used to build a model. Independent variables with an association (*p* < 0.2) with satisfactory QOL outcome in univariate analysis were entered into the model. With backward elimination, risk factors (*p* < 0.2) were kept in the model, as described previously [10, 11]. Calibration of the model was tested using the Hosmer–Lemeshow goodness-of-fit test. The discriminatory capability of the model was assessed using the c-statistic, which is the same as the area under the curve. Correlation analysis was used to investigate the association between weight loss and total score in MAII. Weight loss was expressed as change in BMI (ΔBMI), percent total weight loss (%TWL), and percent excess weight loss (%EWL).

## Results

### General Patient Characteristics

Table 1 presents the descriptive characteristics of the patients. We enrolled 58 patients in the operated group and 101 in the control group. The operated group consisted of 10 women and 18 men after LSG (post-LSG subgroup) and 18 women and 12 men after LRYGB (post-LRYGB subgroup). The control group comprised 64 women and 37 men. The mean age of patients in the operated group was 43.6 ± 10.8 years, including 46.7 ± 10.5 years for the post-LSG subgroup and 40.6 ± 10.5 years for the post-LRYGB subgroup. The mean age of patients in the control group was 40.2 ± 9.1 years. The mean BMI of patients in the operated group was 27.6 ± 2.6, including 28.6 ± 2.7 for the post-LSG subgroup and 26.7 ± 2.2 for the post-LRYGB subgroup. The mean BMI of patients in the control group was 45.1 ± 7.4. The mean ΔBMI in the operated group was 14.8 ± 6.8, including 18.6 ± 6.7 for the post-LSG subgroup and 11.4 ± 4.7 for the post-LRYGB subgroup. %TWL and ΔBMI was significantly higher in post-LSG subgroup. %EWL was comparable in both groups.

**Table 1:** Descriptive characteristics of all patients involved in study

Characteristic	Operated group (*n* = 58)		Control group (*n* = 101)	*p* value
Mean age (years)	43.6 ± 10.8		40.2 ± 9.1	0.12^a^
Gender (F/M)	28/30		64/37	0.07^b^
Mean BMI (kg/m^2^)	After surgery	27.6 ± 2.6	45.1 ± 7.4	<0.01^a^
	Before surgery	45.9 ± 6.7		0.50^a^
ΔBMI (kg/m^2^)	14.8 ± 6.8		–	–
%TWL	31.5 ± 13.6		–	–
%EWL	83.4 ± 19.8		–	–
Comorbidity				
Hypertension	After surgery	4 (7 %)	47 (46 %)	<0.01^b^
	Before surgery	19 (33 %)		0.10^b^
Diabetes	After surgery	0 (0)%	17 (17 %)	<0.01^b^
	Before surgery	11 (19 %)		0.83^b^
Partnered status				
Partnered	53 (91 %)		91 (91 %)	1.0^b^
Non-partnered	5 (9 %)		10 (9 %)	
Smoking status				
Never-smoker	33 (58 %)		38 (39)%	0.01^b^
Current smoker	14 (25 %)		21 (22 %)	
Former smoker	10 (17 %)		38 (39 %)	
Hometown population				
<100,000	1 (2 %)		30 (32 %)	<0.01^b^
100,000–250,000	5 (9 %)		8 (8 %)	
250,000–500,000	9 (16 %)		8 (8 %)	
>500,000	41 (73 %)		50 (52 %)	

*F* female; *M* male; *%TWL* % total weight loss; *%EWL* % excess weight loss

^a^The Mann–Whitney *U* test was used

^b^Fisher’s test was used

The operated and control groups were comparable regarding gender, age, and partnered status. The prevalence of diabetes and hypertension was higher in the control group. There was a higher percentage of never smokers in the operated group. More patients from the control group live in large cities. The post-LRYGB and post-LSG subgroups were comparable regarding gender, partnered status, smoking status, and hometown population. No diabetes was observed in the operated group. There were differences in age, BMI, and hypertension between the post-operative and control groups (Table 2).

**Table 2:** Descriptive characteristics of patients after bariatric surgery

Characteristic		Post-LRYGB subgroup (*n* = 30)	Post-LSG subgroup (*n* = 28)	*p* value
Mean age (years)		40.6 ± 10.5	46.7 ± 10.5	0.03^a^
Gender (F/M)		18/12	10/18	0.07^b^
Mean BMI (kg/m^2^)	After surgery	26.7 ± 2.2	28.6 ± 2.7	<0.01^c^
	Before surgery	38.1 ± 4.3	47.1 ± 6.0	<0.01^c^
%TWL		26.6 1 ± 1.6	38.9 ± 13.9	<0.01^c^
ΔBMI (kg/m^2^)		11.4 ± 4.7	18.6 ± 6.7	<0.01^c^
%EWL		85.1 ± 22.7	81.4 ± 16.2	0.4^c^
Comorbidity				
Hypertension	After surgery	0 (0 %)	4 (14 %)	0.05^b^
	Before surgery	10 (33 %)	9 (32 %)	1.0^b^
Diabetes	After surgery	0 (0 %)	0 (0 %)	–
	Before surgery	9 (30 %)	2 (7 %)	0.04^b^
Partnered status				
Partnered		22 (85 %)	26 (96 %)	0.19^b^
Non-partnered		4 (15 %)	1 (4 %)	
Smoking status				
Never-smoker		19 (63 %)	14 (52 %)	0.62^b^
Current smoker		7 (23 %)	7 (26 %)	
Former smoker		4 (13 %)	6 (22 %)	
Hometown population				
< 100,000		0 (0 %)	1 (4 %)	0.29^b^
100,000–250,000		3 (10 %)	2 (8 %)	
250,000–500,000		7 (23 %)	2 (8 %)	
> 500,000		20 (67 %)	21 (80 %)	

*F* female; *LRYGB* laparoscopic Roux-en-Y gastric bypass; *LSG* laparoscopic sleeve gastrectomy; *M* male; *F* female; *M* male; *%TWL* % total weight loss; *%EWL* % excess weight loss

^a^The two-sample *t* test was used

^b^Fisher’s test was used

^c^The Mann–Whitney *U* test was used

### Preoperative Data of Operated Group

The mean BMI of operated patients before surgery was 45.9 ± 6.7 including 47.1 ± 6.0 for the LSG subgroup and 38.1 ± 4.3 for the LRYGB subgroup. Nineteen patients had hypertension including ten patients in LRYGB subgroup and nine patients in LSG group. Eleven cases had diabetes, including nine patients in LRYGB subgroup and two patients in LSG group. Patients qualified to LRYGB were younger and had a higher percentage of diabetes. Preoperative characteristic of operated group was comparable to controls in terms of BMI, prevalence of diabetes, and hypertension.

### QOL in Control Versus Operated Groups

In the MA II, the total score was 1.70 ± 0.76 in the operated group, which was significantly higher than 0.59 ± 1.17 in the control group (*p* < 0.01). Detailed analysis revealed significantly higher scores in the following areas: general self-esteem, physical activity, social contacts, and focus on eating behavior (Table 3). The QOL outcomes were significantly different between the post-operative and control groups. A satisfactory QOL outcome in MA II (endpoint) was achieved in 48 patients in the operated group and 33 in the control group [odds ratio (OR) 8.89, 95 % confidence interval (CI) 3.98–19.79, *p* < 0.05] (Table 4).

**Table 3:** Comparison of MA II scoring between post-operative and control groups

MA II domains	Scoring		
	Operated group	Control group	*p* value
General self-esteem	0.36 ± 0.14	0.10 ± 0.27	<0.01^a^
Physical activity	0.31 ± 0.14	−0.08 ± 0.30	<0.01^a^
Social contacts	0.33 ± 0.18	0.22 ± 0.26	<0.01^a^
Work satisfaction	0.25 ± 0.19	0.22 ± 0.25	0.97^a^
Sexual pleasure	0.21 ± 0.26	0.12 ± 0.30	0.10^a^
Focus on eating behavior	0.24 ± 0.21	0.00 ± 0.28	<0.01^a^
Total score	1.70 ± 0.76	0.59 ± 1.17	<0.01^b^

*MA II* Moorehead–Ardelt quality of life questionnaire II

^a^The Mann–Whitney *U* test was used

^b^The two-sample *t* test was used

**Table 4:** Comparison of QOL outcomes between control and operated group

Groups*	QOL outcome (*n*)					
	Very poor	Poor	Fair	Good^a^	Very good^a^	Total satisfactory outcomes
Control (*n* = 101)	3	5	53	25	8	33
Postoperative (*n* = 58)	0	0	10	26	22	48

*Fisher’s exact test *p* < 0.01 for distribution of endpoints between post-operative and control groups

*QOL* quality of life

^a^Satisfactory outcomes

### Type of Bariatric Procedures and QOL

The total MA II score in the post-LSG patients was 1.71 ± 0.76 and 1.70 ± 0.77 in the post-LRYGB subgroup. Regarding detailed QOL scoring, there was no difference between patients who underwent LSG or LRYGB (Table 5). The prevalence of different outcomes was similar in both post-surgical groups (Table 6).

**Table 5:** Comparison of MA II scoring between post-operative subgroups

MA II domains	Scoring		
	Post-LRYGB subgroup	Post-LSG subgroup	*p* value
General self-esteem	0.35 ± 0.14	0.36 ± 0.15	0.74^a^
Physical activity	0.31 ± 0.17	0.32 ± 0.12	0.94^a^
Social contacts	0.36 ± 0.17	0.31 ± 0.18	0.19^a^
Work satisfaction	0.23 ± 0.18	0.26 ± 0.20	0.37^a^
Sexual pleasure	0.20 ± 0.23	0.22 ± 0.29	0.56^a^
Focus on eating behavior	0.25 ± 0.23	0.23 ± 0.19	0.47^a^
Total score	1.70 ± 0.77	1.71 ± 0.76	0.94^b^

*LRYGB* laparoscopic Roux-en-Y gastric bypass; *LSG* laparoscopic sleeve gastrectomy; *MA II* Moorehead–Ardelt quality of life questionnaire II

^a^The Mann–Whitney *U* test was used

^b^ *t* test was used

**Table 6:** Comparison of QOL outcomes between post-operative subgroups

Groups*	QOL outcome (*n*)					
	Very poor	Poor	Fair	Good^a^	Very good^a^	Total satisfactory outcomes
Post-LRYGB (*n* = 30)	0	0	6	13	11	24
Post-LSG (*n* = 28)	0	0	4	13	11	24

*LRYGB* laparoscopic Roux-en-Y gastric bypass; *LSG* laparoscopic sleeve gastrectomy; *QOL* quality of life

*Fisher’s exact test, *p* = 0.73, for distribution of endpoints between post-operative subgroups

^a^Satisfactory outcomes

### Factors Associated With Increased Risk of Satisfactory QOL Outcome in MA II

**Fig. 1:**
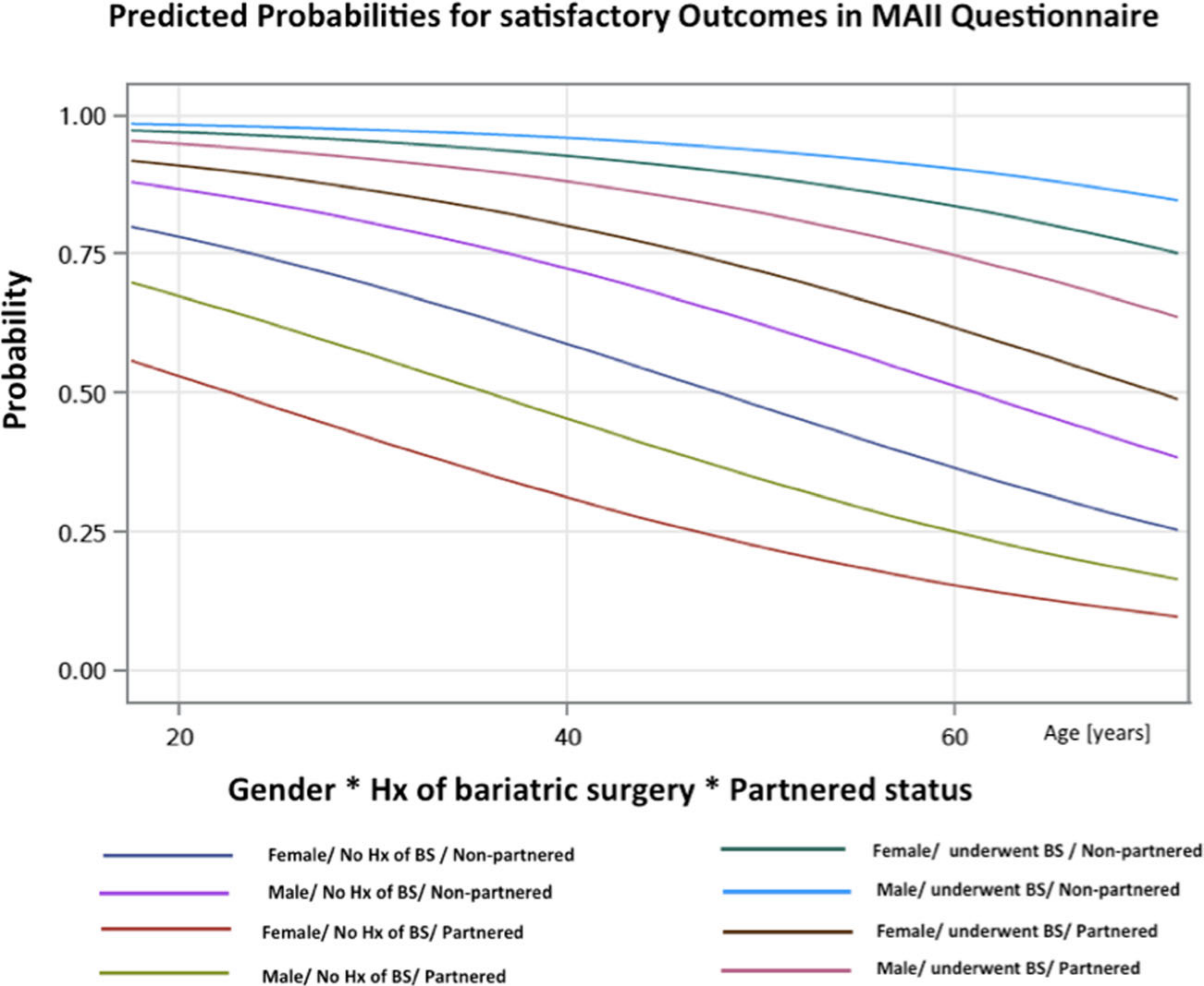
Probabilities for satisfactory outcomes in MA II among obese patients

Data of all 159 patients were analyzed. Of the nine examined variables, only the following were predictive for satisfactory QOL outcome in multivariate analysis: age (OR 0.955; 95 % CI 0.911–1.002), female gender (OR 0.546; 95 % CI 0.241–1.234), no history of bariatric surgery (OR 0.113; 95 % CI 0.044–0.290), and non-partnered status (OR 3.154; 95 % CI 0.752–13.222) (Table 7). Figure 1 presents predicted probability for different groups of patients. The multiple logistic regression equation was as follows: L = 3.001 + (−0.046 × Age) + (−0.303 × Female) + (−1.092 × No history of bariatric surgery) + (0.574 × Non − partnered). The model presented good discrimination (c-statistics 0.773) and good calibration (Hosmer–Lemeshow goodness-of-fit test, χ^2^ = 4.418, *p* = 0.817).

**Table 7:** Factors for satisfactory QOL outcome in MA II

Factors	Adjusted OR	95 % wald CI	Estimate	Standard error	Wald *χ* ^2^	*p* value
Constant			3.001	1.1994	6.2599	0.0124
Age	0.955	0.911–1.002	−0.046	0.0243	3.5180	0.0607
Female	0.546	0.241–1.234	−0.303	0.2081	2.1165	0.1457
No Hx of bariatric surgery	0.113	0.044–0.290	−1.092	0.2418	20.4056	<0.0001
Non-partnered	3.154	0.752–13.222	0.574	0.3656	2.4678	0.1162

*MA II* Moorehead–Ardelt quality of life questionnaire II; *QOL* quality of life

### Correlation Between Weight Loss and Total Score in MAII

The data of 58 operated patients were analyzed. No significant correlation was present between total score in MAII and the following psychometric variables: %TWL (*r* = −0.122, *p* = 0.36), ΔBMI (*r* = −0.107, *p* = 0.42), and %EWL (*r* = −0.064, *p* = 0.63).

## Discussion

In our study, total MA II score was significantly higher in the operated than control group. Also, the percentage of patients with satisfactory QOL outcomes was significantly higher in the operated group, which reflected better QOL. However, in patients after surgery, the scoring adjusted by type of surgery was comparable.

Among nine examined variables, age, gender, history of bariatric surgery, and partnered status had an influence on QOL. History of surgery had the strongest independent association with the probability of satisfactory QOL outcome. Our model had good calibration. The discriminative ability of the model was above the cutoff of 0.70. Thus, the proposed model has clinical value. On the basis of the regression equation and parameters presented in Table 7, it is possible to calculate probability of satisfactory QOL outcome in obese individuals, depending on whether they have had bariatric surgery. The study revealed that age, gender, and partnered status were confounders for QOL outcome and should be take into account in further studies about QOL and bariatric surgery. Surprisingly, BMI was not associated with QOL score. Moreover, in the detailed analysis of post-operative patients we found that the body mass reduction was not correlated with total score in MAII.

Knowledge about changes in QOL after surgical weight loss is essential for every bariatric surgeon. In 2003, Ballantyne et al. reported a general improvement in QOL after bariatric surgery [12]. However, the trends in bariatric surgery have changed and new procedures like LSG and LRYGB have gained popularity. These procedures are equal in terms of excess weight loss but have a different influence on morbidity. LRYGB seems to be more effective in improving glycemic control and resolving hypertension [4]. The indications for the particular type of procedure are not established and in Poland every center has its own policy.

Significant differences in QOL improvements are found between different types of bariatric surgery [1]. Considering the observed trends in bariatric surgery, there is a need for new studies comparing QOL outcome after LSG and LRYGB—the most commonly performed bariatric operations [3]. In the literature, there is little about the difference between these two procedures in terms of QOL.

Our results corresponded to findings reported by Zangh et al. [5] and Major et al. [6]. It is important to note that these authors used the same questionnaire for the assessment of QOL as we did. Also, the study by Peterli et al., where the authors used the Gastrointestinal Quality of Life Index (GQLI), revealed no difference between post-LRYGB and post-LSG patients [7]. Thus, we provided further evidence that both procedures are equal in terms of QOL improvement.

Many believe that weight loss reflects a successful outcome after bariatric surgery. However, in our study, QOL was not dependent on BMI. Likewise, Müller et al. analyzed factors influencing QOL in 104 patients and found no correlation between BMI and QOL [13]. Furthermore, we found that the QOL outcome in MAII was not dependent on %TWL, %EWL, and ΔBMI. Major et al. stated that QOL was not dependent on percentage of excess weight loss (EWL%) [6]. Their results were similar to those of Sarwer et al., which showed no correlation between body mass reduction and improvement in QOL [14]. We should open a discussion about what is responsible for good QOL outcome after bariatric surgery.

### Limitations

We are aware that our study had several limitations. First, the sample size was small. It has to be pointed out that small populations do not give a strong insight. Small sample size resulted from the low response rate in operated group. This is the major flaw of the study. Sensitive nature of the item referring to sexual life in MAII may have contributed to low compliance in the operated group. Unfortunately, distributing questionnaires via e-mails turned out to be ineffective. Face-to-face collecting data resulted in higher response rate.

The present study was a cross-sectional and we did not use matching in the study. Thus, there were some differences in the characteristics of the analyzed groups. Smoking status and hometown population may be confounding factors and should be considered in future studies.

Last, the period of 12–18 months of observation was short. There is a need for long-term observation studies to assess long-term effects of bariatric surgery on the quality of life.

## Conclusion

This study demonstrates that patients after bariatric surgery have a higher score in the MA II, which reflects better QOL. However, the scoring adjusted for the type of operation is comparable. QOL among obese patients is dependent on age, gender, history of bariatric surgery, and partnered status. Weight loss was not associated with better outcome in MAII.
